# Supplementary material for: The Anti-Acne Potential and Chemical Composition of Two Cultivated Cotoneaster Species
Source: Cells. 2022 Jan 21;11(3):367. doi: 10.3390/cells11030367 (PMC8834067; doi:10.3390/cells11030367)
Supplement: Supplementary file 1 [file cells-11-00367-s001.zip › cells-1512437-supplementary.pdf]

cells

MDPI

Supplementary Materials

Table S1. Zones of bacterial growth inhibition of the Cotoneaster extracts and fractions.

Sample	*C. acnes* PCM 2334	*C. acnes* PCM 2400	*C. acnes* ATCC 11827	*C. granulosum* PCM 2462	*S. aureus* ATCC 25923	*S. epidermidis* ATCC 12228	*P. aeruginosa* ATCC 27853	*E. coli* ATCC 25992
CHi	12 ± 1	11 ± 3	10 ± 3	6 ± 1	8 ± 2	6 ± 1	0	0
CHs	10 ± 2	12 ± 2	12 ± 2	6 ± 2	10 ± 2	6 ± 2	0	0
CHs-1	15 ± 2	15 ± 1	14 ± 3	14 ± 3[a,b]	13 ± 2	12 ± 2[a,b]	0	0
CHs-2	20 ± 2[a,b]	23 ± 1[a,b,c]	22 ± 1[a,b,c]	21 ± 1[a,b]	17 ± 2[a,b]	19 ± 1[a,b,c]	0	0
CHs-3	14 ± 3[d]	11 ± 3[d]	12 ± 3[d]	13 ± 2[a,b]	6 ± 3[c,d]	8 ± 2[d]	0	0
CHs-4	16 ± 1[b]	18 ± 3[a]	19 ± 3[a]	17 ± 2[a,b]	17 ± 3[a,b,e]	15 ± 1[a,b,e]	0	0

Statistical analysis: a—significantly different results compared to CHi; b—significantly different results compared to CHs; c—significantly different results compared to CHs-1; d—significantly different results compared to CHs-2; e—significantly different results compared to CHs-3; One-Way ANOVA test, followed by a Tukey's multiple comparison test, $p < 0.05$. CHi—crude extract of *C. hissaricus*, CHs—crude extract of *C. hsingshangensis*, CHs-1—water fraction of *C. hsingshangensis*, CHs-2—diethyl ether fraction of *C. hsingshangensis*, CHs-3—butanol fraction of *C. hsingshangensis*, CHs-4—ethyl acetate fraction of *C. hsingshangensis*.
